# CNS resident macrophages enhance dysfunctional angiogenesis and circulating monocytes infiltration in brain arteriovenous malformation

**DOI:** 10.21203/rs.3.rs-2899768/v1

**Published:** 2023-05-12

**Authors:** Li Ma, Xiaonan Zhu, Chaoliang Tang, Peipei Pan, Alka Yadav, Rich Liang, Kelly Press, Hua Su

**Affiliations:** University of California, San Francisco; University of California, San Francisco; University of California, San Francisco; University of California, San Francisco; University of California, San Francisco; University of California, San Francisco; University of California, San Francisco; University of California, San Francisco

**Keywords:** Arteriovenous malformation, Macrophages, Angiogenesis, CSF1R inhibitor

## Abstract

Myeloid immune cells present abundantly in both ruptured and unruptured brain arteriovenous malformations (bAVMs). The role of central nervous system (CNS) resident and circulating monocyte-derived macrophages in bAVM pathogenesis has not been fully understood. RNA sequencing using cultured cells and bAVM samples revealed that downregulation of activin-like kinase 1 (*ALK1*) or endoglin (two bAVM causative genes) increased pro-angiogenic, endothelial inflammation and innate immune signaling, which provided endogenous underpinnings of the active inflammation in bAVM. To further understand the role of CNS resident macrophages in bAVM development and hemorrhage, we administrated a colony-stimulating factor 1 receptor (CSF1R) inhibitor to bAVM mice with endothelial *Alk1* deletion. Transient depletion of CNS resident macrophages at early stage of bAVM development remarkably mitigated the subsequent phenotype severity of bAVM. This therapeutic effect exhibited a prolonged inhibition of angiogenesis, dysplastic vasculature formation, and infiltration of CNS resident and circulating monocyte-derived macrophages during bAVM development. Transient depletion of CNS resident macrophages also reduced the dysplasia vessels and improved the integrity of endothelial tight junctions in established bAVMs. Administration of CSF1R inhibitor also prevented severe hemorrhage of bAVMs. Thus, endothelial AVM causative gene mutation can activate CNS resident macrophages promoting bAVM progression. CNS resident macrophages could be specific targets to mitigate the development and severity of bAVMs.

## Introduction

Arteriovenous malformations (AVMs) are active angiogenic lesions consisting of tangles of abnormal vessels shunting blood directly from arteries to veins without a true capillary bed [1]. Innate immune response in bAVM is characterized by macrophage infiltration. An abnormally high number of macrophages are present in and around vascular walls in human brain AVM (bAVM) specimens, with or without hemorrhage, suggesting that macrophage accumulation is not simply a response to hemorrhage [2–6]. Polymorphisms in inflammatory cytokines and elevated expressions of inflammation-related genes in AVM patients further suggest their active roles in bAVM pathogenesis [7–9]. We have found increased macrophage burden in bAVMs in mouse models generated through conditional deletion of endoglin (*Eng*) or activin-like kinase 1 (*Alkl*, also known as *ACVLR1*), in combination with focal angiogenic stimulation [10–13]. *ENG* and *ALK1* are causative genes of hereditary hemorrhagic telangiectasia (HHT), which is a familial disorder with high prevalence of bAVMs [14, 1]. The activated macrophages were also found abundantly in bAVM mouse model with endothelial KRAS gain of function, which is a somatic mutation identified in sporadic bAVMs [15]. Macrophage accumulation in bAVM represents unresolved inflammation, which then can enhance abnormal vascular remodeling and the severity of the bAVM phenotype. It is unclear whether these known gene mutations directly contribute to neuroinflammation in bAVMs. Understanding the mechanism of macrophage accumulation may afford an opportunity to improve bAVM patient care [6].

In addition, persistent proinflammatory differentiation of macrophage has been found to be critical in bAVM progression. We identified both the central nervous system (CNS) resident and circulating myeloid cells as potential precursors of proinflammatory macrophages in bAVM nidus [16]. Recent studies of human bAVM tissues unraveled the crosstalk between immune cells and vascular components [17]. More importantly, a heterogenous spectrum of myeloid cells with typical molecular signature and spatial distribution were clustered, which are distinct in ruptured bAVM and associated with instability of AVM vasculature. The resident myeloid cells were the major immune cells infiltrating along the perivascular space and deeper brain tissue, while some specific monocytes were over-represented in ruptured AVMs. It is undetermined which cell clusters act as initiators and which ones respond subsequently. Moreover, potential interplay between CNS endogenous and circulating myeloid cells is yet to be determined. Therefore, further exploration is needed to elucidate the distinct role of myeloid cell subsets and catalog more specific therapeutic targets in bAVM.

Circulating monocytes can infiltrate the CNS and differentiate into macrophages in brain vascular disorders [18]. The recruitment of monocytes into tissues, including CNS, depends on monocyte chemoattractant protein (CCL2) and its receptor (CCR2). We found a delayed but persistent accumulation of CCR2^+^ cells in the bAVM lesions of mouse model [16]. CNS resident macrophages are established in the CNS since embryo and maintain their population independently of circulating monocytes. There are two major clusters of CNS resident macrophages described based on anatomical location, morphology and molecular signatures: microglia (parenchymal specific macrophages) and non-parenchymal macrophages (further classified as perivascular, subdural meninges and choroid plexus macrophages) [19–21]. The ionized calcium-binding adaptor molecule 1 (Iba1), CX_3_CR1, and colony stimulating factor 1 receptor (CSF1R) markers are expressed across all of the CNS resident populations [19]. The stimulation from CSF1R is critical for the development and maintenance of CNS resident macrophages [22]. Pharmacological inhibition of CSF1R depletes Iba1^+^ and CX_3_CR1^+^ cells by 94–98% in parenchyma and ~ 70% in perivascular space [23]. The CSF1R inhibitor, PLX5622, depletes CNS resident myeloid cells, without interfering the infiltration circulating CCR2^+^ monocytes [18, 24]. Therefore, use of PLX 5622 will allow us to understand the role of CNS resident macrophages.

In this study, we demonstrated the endothelial mutation of AVM causative genes can upregulate neuroinflammatory signaling pathways, and CNS resident macrophages actively involved in bAVM pathogenesis.

## Methods

### Animals

8- to 10-week-old *Alk1*^f/f^;*Ccr2*^*RFP/+*^*Cx3cr1*^GFP/+^ mice in C57BL background with two alleles of *Alk1* gene (exons 4 to 6) flanked by loxP sites [25], red fluorescent protein (RFP) gene knocked into one allele of *Ccr2* gene, and green fluorescent protein gene (GFP) knocked into one allele of *Cx3cr1* gene [26] and *Pdgfb*CreER; *Eng*^f/f^ mice with two alleles of *Eng* gene (exons 4 to 5) flanked by loxP sites[27] were used. Equal numbers of male and female mice were included.

Experimental procedures for using laboratory animals were approved by the Institution of Animal Care and Use Committee of the University of California, San Francisco.

### Induction of bAVM through stereotactic injection of viral vector

Brain AVMs were induced in *Alk1*^f/f^;*Ccr2*^*RFP/+*^*Cx3cr1*^GFP/+^ mice through stereotactic intracerebral injection of viral vectors as described in our previous paper[10]. Mice were anesthetized through inhalation of 4% isoflurane and placed in a stereotactic frame with a holder (David Kopf Instruments, Tujunga, CA). A burr hole was drilled in the pericranium 2 mm lateral to the sagittal suture and 1 mm posterior to the coronal suture. A total of 2 μL viral vector suspension containing 2×10^9^ genome copies (gc) of AAV-VEGF (an adeno-associated viral vector expressing human vascular endothelial growth factor) and 2×10^7^ plaque-forming units (PFU) of Ad-Cre (an adenoviral vector carrying CMV promoter driving Cre recombinase expression) were injected into the basal ganglia 3 mm beneath the brain surface. Ad-GFP and AAV-LacZ were used as control for Ad-Cre and AAV-VEGF, respectively. Mice were randomly assigned to each treatment groups using flipping a coin method.

Brain AVMs were induced in *Pdgfb*iCreER; *Eng*^f/f^ mice through intra-peritoneal injection of tamoxifen (TM, 3 doses of 2.5 mg/25g of body weight) and intra-brain injection of AAV-VEGF (2×10^9^ genome copies). Control mice were treated with 3 doses of corn oil and intra-brain injection of AAV-VEGF. Brain AVM tissues were collected 8 weeks after model induction. Total RNAs were isolated form bAVM lesions and brain angiogenic regions (controls) for sequence.

### ALK1 knockdown and RNA sequence (RNAseq)

Human umbilical cord endothelial cells (HUVECs) were purchased from (ATCC, Manassas, VA) and cultured in Vascular Cell Basal Medium supplemented with endothelial cell growth factors and VEGF in Endothelial Cell Growth Kits provided by ATCC. Cells within 6 passages were used. To knockdown *ALK1*, 180 pmol of *ALK1* siRNA (Sequence: 5’-CCCUCUACGACUUUCUGCA-3’) [28] custom synthesized by Thermo Fisher Scientific (South San Francisco, CA) were transfected into HUVECs using FlexiTube siRNA purchased from QIAGEN (Hilden, Germany) in RNAiMax (Invitrogen, Waltham MA), according to the instructions of the manufacturers. Scrambled siRNA transfected cells were used as control. Total RNAs were extracted from the cells 48 hours after the transfection using RNAzol RT (Molecular Research Center, Cincinnati, OH).

To check the knockdown efficiency, the RNAs were reverse-transcribed into cDNA using Superscript III First-Strand Synthesis System (Invitrogen, Carlsbad, CA). Real-time PCR was performed using TaqMan Fast Advanced Master Mix (Applied Biosystems, Foster City, CA). Gene-specific primers and probes purchased from Applied Biosystems were used: *ALK1* (Hs00953798_m1), and *GAPDH* (Hs02758991_g1). The relative gene expression was calculated using the comparative threshold cycle (CT) and normalized to *GAPDH* (ΔCT).

Total RNAs isolated from HUVECs with *ALK1* downregulated more than 80%, and from bAVMs and brain angiogenic region of control mice were sent to Novogene Co (Davis, CA) for sequence using the company’s standard protocol (Supplemental material 1). The outcome data were also analyzed by Novogene Co.

### Administration of inhibitor of CSF1R (PLX5622)

PLX5622 (180 mg/kg/day of body weight, Plexxikon Biotech Company, South San Francisco, CA) was incorporated in chow and oral administered for 7 days starting at 1 week or 8 weeks after model induction. The placebo chow was administrated in the same pattern to the control group.

### Quantitative assessment of vessel morphology and macrophages

Brain samples were collected 8 weeks or 9 weeks after model induction. After being anesthetized with isoflurane inhalation, Cy5-fluorescein-conjugated lycopersicon esculentum lectin (Vector Laboratories, Burlingame, CA) was injected via jugular vein to stain endothelial cells. Mice were then perfused with heparinized PBS through left cardiac ventricle to clear blood from vasculature followed by 4% paraformaldehyde. Brain samples were collected and incubated in 4% paraformaldehyde containing 20% sucrose until they sunk to the bottom of the solution. Brain samples were then snap-frozen in dry ice and sectioned.

Coronal sections (20-μm-thick) were cut using a cryostat (Leica, CM1900, Germany). Two coronal sections from each mouse were chosen, 0.5 mm anterior and 0.5 mm posterior to the needle track. Sections were coverslipped with Vectashield mounting medium with 4’-6-diamidino-2-phenylinidole (DAPI, Vector Laboratories) to label cell nuclei. Three images were taken under 20 x objective field of each section (left, right, and below the injection site) using a fluorescent microscope (Keyence BZ-9000, Itasca, IL).

Vascular density (number of vessels per mm^2^), RFP^+^ and GFP^+^ cells (per mm^2^) and claudin 5^+^, CD31^+^ vessels were quantified in using NIH Image 1.63 software. Dysplasia vessels were counted manually. Dysplasia index (the number of vessels with lumen diameter larger than 15 μm/mm^2^) were used to express the quantify of dysplasia vessels.

### Prussian blue staining

Iron Stain Kit (Sigma-Aldrich, St. Louis, MO) was used to detect iron deposition. Slides were incubated in freshly prepared working iron stain solution for 15 minutes, washed in distilled water, and counterstained with pararosaniline solution for 3 minutes. Two sections per brain within the injection site were chosen for staining. Data presented as percentage of Prussian blue positive area in the hemisphere.

### Statistics

For quantification of vessels density, dysplasia index, RFP^+^ and GFP^+^ cell numbers, vascular claudin 5 coverage and Prussian blue staining areas, section numbers were scrambled. The quantification was done by two researchers blinded to the treatment groups independently. Inter-observer discrepancy was controlled within one standard deviation, and the means were used for further analysis. Data are presented as mean ± standard deviation (SD). All data were analyzed through t test for two sample-comparation, or one-way ANOVA for multiple sample-comparation followed by Tukey’s multiple comparisons using GraphPad Prism 9 software. A *P* value < 0.05 was considered to be significant. Sample sizes were indicated in figures.

## Results

### Downregulation of ALK1 or Eng in endothelial cells upregulated pro-inflammatory and innate immune signaling.

**Figure 1:**
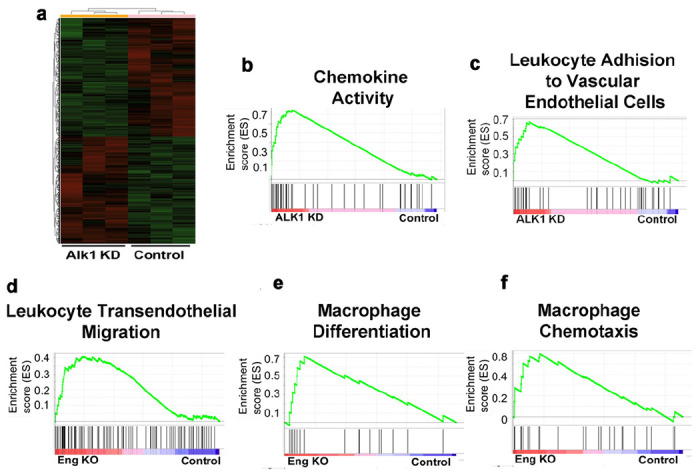
Down regulation of *ALK1* in HUVECs or *Eng* in mouse bAVM increased pro-inflammation signaling. **a**. A heat map shows differential gene expression in *ALK1* siRNA and scrambled siRNA treated HUVECs. **b and c.** Increased signaling of chemokine activity and leukocyte adhesion to vascular endothelial cells in *ALK1* siRNA treated HUVECs. **d, e, and f** show increased signaling of leukocyte transendothelial migration, macrophage differentiation and macrophage chemotaxis in *Eng* deficient bAVMs. ALK1 KD: HUVECs with *ALK1* gene knocked down; Control (in c): scrambled siRNA treated HUVECs; Eng KO: mice with Eng deletion in endothelial cells; and Control (in d, e, f): corn oil treated mice.

To understand the roles of *Alk1* and *Eng* genes in endogenous angiogenesis and inflammation, we knocked down *ALK1* in HUVECs and knocked out *Eng* in mouse endothelial cells. The transcriptional profiles of *ALK1* deficient HUVECs, brain angiogenic regions of *Eng* deficient mice (bAVMs) and control mice were analyzed by RNAseq. We found that down regulation of *ALK1* in HUVECs upregulated the expression of 507 genes and downregulated the expression of 563 gene compared to scrambled siRNA treated HUVECs. The gene transcriptional profiles are distinctive between *ALK1* down regulated and control HUVECs (Fig. 1a). Gene Ontology (GO) enrichment analyses showed that knockdown of *ALK1* in HUVEC increased the transcription of genes regulating angiogenesis, innate immune response and chemokine-mediated signaling pathway, including neutrophil and monocyte chemotaxis, interleukin-1 beta production and secretion, and tumor necrosis factor production (Figs. 1b & 1c).

We have also analyzed the changes of gene transcription in bAVMs of mice with *Eng* deleted in endothelial cells. We found 1243 genes were upregulated, and 1830 genes were downregulated in bAVM, compared to controls (brain angiogenic region of corn oil treated mice). GO enrichment and Kyoto encyclopedia of genes and genomes (KEGG) analyses showed that *Eng* endothelial deficiency increased the transcription of genes upregulating angiogenesis, leukocyte transendothelial migration, macrophage differentiation and macrophage chemotaxis (Fig. 1d–1f). GO enrichment analyses also show that *Eng* endothelial deficiency increased the transcription of gene sets related to myeloid leukocyte differentiation and migration, myeloid leukocyte activation, phagocytosis, activation and regulation of innate immune response, neutrophil chemotaxis, myeloid cell development, positive regulation of chemotaxis, glial cell proliferation and migration, phagocytic vesicle. Differential analysis shown that the transcription of *Cx3cr1* (adjusted *P* < 0.001) and colony stimulating factor 1 (*Csf1*) gene (adjusted *P* = 0.003) were increased significantly in the bAVM of *Eng* endothelial deficient mice.

These data indicate that down regulation of *ALK1* or *Eng* expression in endothelial cells increases endothelial cell inflammation, leukocyte extravasation from vessels, and activates tissue resident macrophages.

### Transient depletion of CNS resident macrophages reduced the burden of Cx3cr1^+^ and Ccr2^+^ macrophages in bAVM

**Figure 2:**
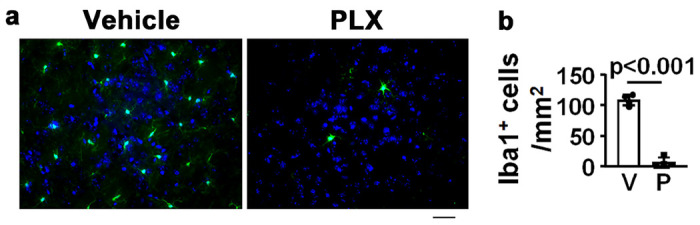
CSF1R inhibitor reduced CNS resident macrophages in wild type mouse brain. **a**. Microscopic images of Iba1 antibody-stained sections collected 7 days after PLX5622 (PLX) treatment. Iba1^+^ cells stained green. The nuclei were stained by DAPI (blue). Scale bar =30 mm. **b.** Quantification of Iba1^+^ cells. V: vehicle treated; P: PLX5622 treated mice. N=4.

To investigate the specific function of CNS resident macrophage in bAVM inflammation, Cx3cr1^+^ CNS resident macrophages were transiently depleted through oral administration of PLX5622. We first tested the efficiency of PLX5662 on depletion of CNS resident macrophages by administration of it to wild-type (WT) mice for 7 days. We found 93% of Iba1^+^ cells were depleted in the brains of treated mice (Fig. 2).

**Figure 3:**
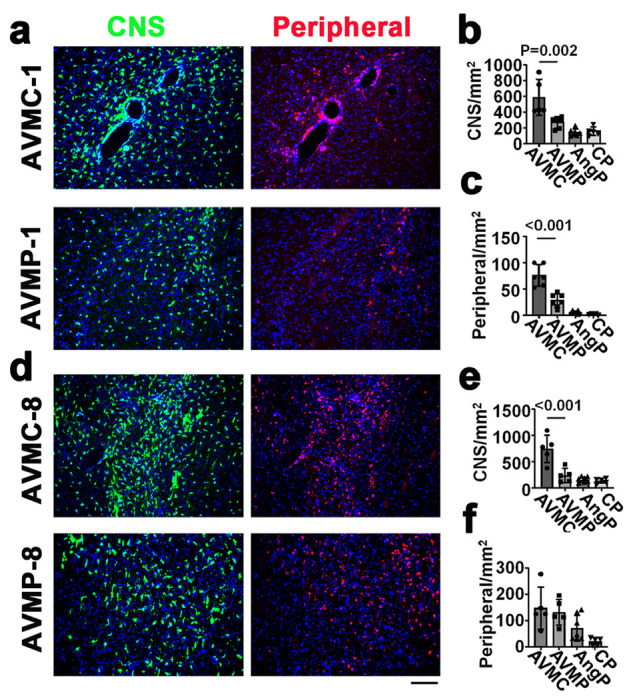
PLX5622 treatment reduced CNS resident and circulating monocyte-derived macrophages in mouse bAVMs. **a & d.** Representative images of bAVMs collected from mice received PLX5622 starting 1 week (**a**) and 8 weeks (**d**) after model induction. CNS resident macrophages (CNS) are GFP^+^ (green) and peripheral monocyte-derived macrophages (Peripheral) are RFP^+^ (red). Nuclei were counterstained with DAPI (blue). Scale bar= 50μm. **b & c.** Quantifications of CNS resident macrophages (**b**) and peripheral monocyte-derived macrophages (**c**) in the bAVMs of mice received PLX5622 starting 1 week after model induction. **e & f**. Quantifications of CNS resident macrophages (**e**) and peripheral monocyte-derived macrophages(**f**) in the bAVMs of mice received PLX5622 8 weeks following model induction. AVMC: AVM mice treated with vehicle; AVMP: AVM mice treated with PLX 5622; AngP: mice with brain angiogenesis and PLX5622 treatment; CP: mice received intra-brain injection of control vectors PLX5622 treatment. N=5-7.

We next administrated PLX5662 in mice with bAVMs generated by focal deletion of *Alk1* gene plus angiogenic stimulation [10] to test the effect of transient depletion of CNS resident macrophage on bAVM pathogenesis. PLX 5622 treatment for 7 days starting either at 1 week after model induction when the bAVM development begun (*P* = 0.002) or 8 weeks after model induction when bAVMs have established (*P* < 0.001) reduced *Cx3cr1*^+^ resident macrophages in bAVM lesions (Fig. 3).

Administration of PLX 5622 starting at 1 week after model induction when bAVM development begun also reduced circulating monocyte-derived macrophages (*Ccr2*^+^) in bAVMs (*P* < 0.001 compared to controls) (Fig. 3). However, PLX 5622 treatment did not reduce the number of circulating monocyte-derived macrophages in established bAVMs. Therefore, transient depletion of resident macrophages at early stage of bAVM development can reduce circulating monocytes infiltration into bAVM lesion, suggesting a pivotal role of resident macrophage in bAVM inflammation.

### Transient depletion of CNS resident macrophages at the beginning of bAVM development reduced angiogenic activity in bAVMs

**Figure 4:**
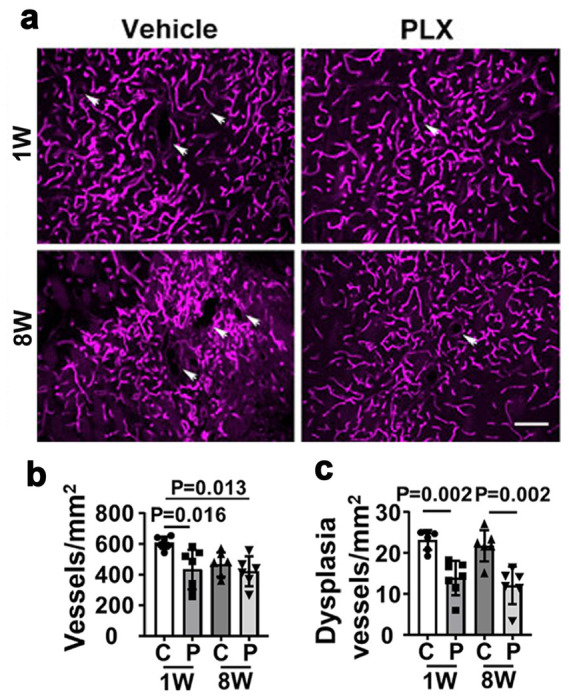
PLX5622treatment reduced dysplastic vessels in mouse bAVMs. **a.** Representative images of brain sections collected from lectin-perfused mice. Vessels are labeled by violet fluorescence. White arrows indicate dysplastic vessels. Scale bar= 50μm. **b.** Quantifications of vessel density. **c.** Quantification of dysplasia index (number of vessels with lumen diameter >15 mm/mm2. C: vehicle treated. P: PLX5622 treated. 1W and 8W: treatment started at 1 and 8 weeks after model induction. N=6 or 7.

To explore the role of CNS resident macrophages on bAVM angiogenesis, we assessed the blood vessel densities within bAVMs. The vessel densities of bAVMs in mice treated with PLX5622 at the beginning of bAVM development (434.1 ± 128.9 vessels/mm^2^) were lower than that in vehicle-treated mice (609.4 ± 40.5 vessels/mm^2^, *P* = 0.016) (Fig. 4). However, the vessel densities were similar in the bAVMs of mice treated with PLX5622 and vehicle 8 weeks after model induction (Fig. 4). Therefore, transient depletion of CNS resident macrophages at the early stage of bAVM development reduced the angiogenic activity, and the effect persistent after the treatment has stopped.

### Transient depletion of CNS resident macrophages alleviated bAVM severity.

**Figure 5:**
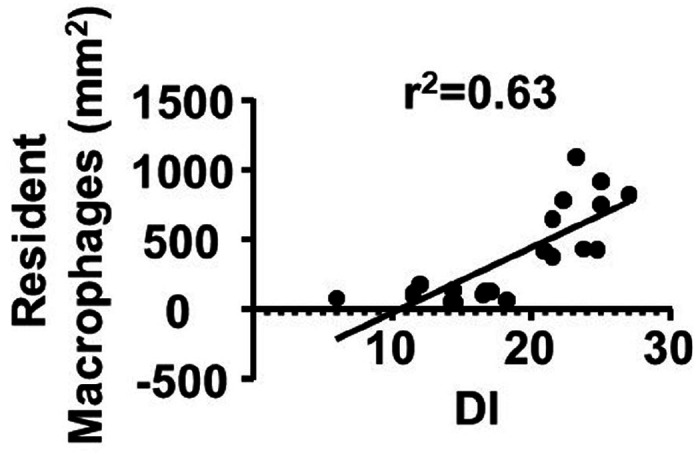
The number of CNS resident macrophages is positively corrected with the number of dysplasia vessels in bAVM. DI: dysplasia index.

We next evaluate the effect of PLX5622 treatment on bAVM severity by examining the abnormal vessels with dilated and irregular lumen. PLX5622 treatment starting at 1 week after the model induction inhibited bAVM development. Even though the PLX5622 treatment had stopped for 6 weeks, there were still fewer dysplasia vessels in treated group (13.9 ± 4.2 vessels/mm^2^) than control group (23.1 ± 2.4 vessels/mm^2^, *P* = 0.002). Administration of PLX5622 starting at 8 weeks after the model induction when bAVMs have already established also reduced the number of abnormal vessels (treated: 12.0 ± 4.6 vessels/mm^2^ vs. control: 21.6 ± 3.9 vessels/mm^2^, *P* = 0.002) (Fig. 4). Notably, the number of *Cx3cr1*^+^ CNS resident macrophages was positively correlated with the number of abnormal vessels in the bAVMs (DI, r^2^ = 0.63, *P* < 0.01, Fig. 5). Taken together, these data show that transient depletion of CNS resident macrophage can not only exhibit a long-term effect of ameliorating bAVM development but also reduce the severity of established bAVMs.

### Transient depletion of CNS resident macrophages enhanced blood brain barrier (BBB) integrity and attenuated hemorrhage in bAVMs

**Figure 6:**
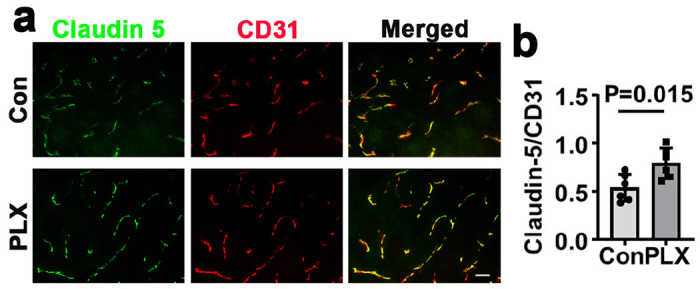
PLX5622 treatment increases claudin 5 expressions in bAVM vessels. **a.** Representative images of sections stained with anti-claudin 5 (green) and CD31 (endothelial cells, red) antibodies. Scale bar = 30μm. **b.** Quantification of the ratio of claudin-5 and CD31. Con: vehicle treated mice; PLX: PLX5622 treated mice. N=6.

Our previous study revealed impairment of vascular integrity in bAVMs [11]. To explore the impact of CNS resident macrophages on BBB integrity of established bAVM, the expression of tight junction protein was quantified. We found PLX5622 treatment restored the expression of claudin 5 in established bAVM (Fig. 6), suggesting that transient depletion of CNS resident macrophage can rescue abnormal vessels and BBB integrity independently from circulating monocyte infiltration.

**Figure 7:**
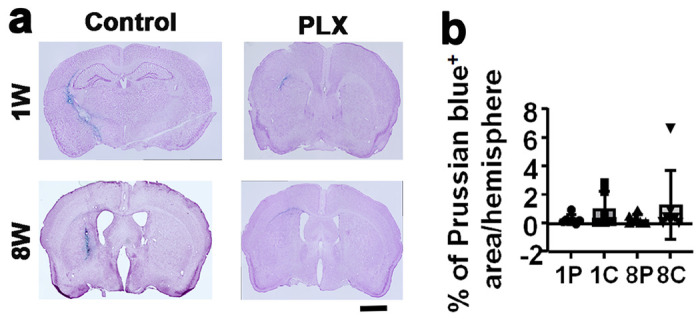
PLX5622 treatment reduced the risk of severe hemorrhage in bAVMs. **a.** Representative images of Prussian blue stained sections. The iron depositions (blue) in bAVMs indicates hemorrhage. The nuclei were counterstained with Fast Red. Scale bar=1mm. **b.** Quantification of Prussian blue-positive area (% of Prussian blue area in corresponding hemisphere). 1W and 8W: treatment started at 1 and 8 weeks after model induction. 1P and 8P: mice treated with PLX5622 starting at 1 week and 8 weeks after model induction; 1C and 8C: mice treated with vehicle starting at 1 week and 8 weeks after model induction. N=5-6.

We further tested whether transient depletion of CNS resident macrophage reduce or prevent bAVM hemorrhage. Hemorrhage in bAVMs were detected by analyzing iron deposition using Prussian staining. PLX5622 treatment starting at 1 or 8 weeks after model induction prevented severe hemorrhage (Prussian blue staining area > 1% of total hemisphere). No mouse in treated groups had hemorrhage area lager that 1% of total hemisphere, the average hemorrhage areas were 0.28% for mice received the treatment starting at 1 week and 0.29% for mice received the treatment starting at 8 weeks after model induction. In contrast, the hemorrhage was more severe in controls. Two out of six mice in 1-week vehicle group and two out of seven mice in 8-week vehicle group have hemorrhage area over 1% of total hemisphere (Fig. 7).

## Discussion

Our findings demonstrated that the genetic dysfunction of *ENG* and *ALK1* in endothelial cells promote transendothelial leukocyte extravasation and activate innate immune system. Notably, CNS resident macrophages play an important role in promoting the development of bAVM by enhancing abnormal angiogenesis and infiltration of both resident and circulating inflammatory cells. Through pharmacological depletion of CNS resident macrophages transiently at different stages of bAVM development, we were able to investigate the temporal role of resident macrophages in bAVM development. In the initial stage of bAVM development, CNS resident macrophages enhance abnormal angiogenesis and the recruitment of blood-borne monocytes into the angiogenic foci. During the time window preceding the infiltration of Ccr2^+^ circulating cells, a transient suppression of CNS resident macrophages reduced the recruitment of Ccr2^+^ circulating cells and mitigated the development of bAVM. The effect extended 6 weeks after ceasing PLX5622 treatment. In the established bAVMs with abundant dysplastic vessels and inflammatory cells, CNS resident macrophage continued to play a critical role in inducing vascular impairment and hemorrhage. Short-term treatment with the PLX5622 in mice with the ‘mature’ bAVMs, that are more approximate to the clinical scenario, has restored dysplasia vessels towards normal phenotype and improved vascular integrity, which ultimately lead to reduction of severe hemorrhage.

Although several germline and somatic mutations have been reported in human bAVMs, the mechanism by which these mutations endogenously amplify the dysfunctional angiogenesis and maintain inflammation during bAVM development, is not fully understood. Recent studies suggested that clonal expansion of mutant endothelial cells contributes to this process [29]. However, the burden of pathogenic mutations is not consistently associated with the phenotype severity of human bAVMs, which are typically heterozygotes or somatic mosaicism [30]. Therefore, the synergistic cofactors upon genetic mutation have yet to be identified. The present study highlights the temporal modulating role of CNS resident macrophages in abnormal angiogenesis and inflammation during bAVM development.

Excessive and dysfunctional angiogenesis is a key process in bAVM development. Increased levels of VEGF and its receptors were found in human bAVM tissue. The single-cell profile of human bAVM tissue identified a differentiation trajectory of endothelial cells to angiogenic clusters, which enriched with pro-angiogenic and pro-permeability gene transcription. These endothelial cells were predicted to be the major contributors of dysregulated angiogenic communication network in bAVM, including angiopoietin, VEGF, TGF-b signaling [17]. Here, we provided direct evidence, both *in vitro* and *in vivo*, that the *ALK1* and *ENG* gene deletion in endothelial cells could endogenously activate pro-angiogenic signaling. The KRAS^G12V^-induced bAVM also revealed increased VEGF-A mRNA expression and phosphorilated-VEGFR2 which confined to the KRAS mutant endothelial cells [15]. Our previous data indicated a higher proliferation of genetic aberrant endothelial cells than the wild-type endothelial cells in the early stage of bAVM development [29]. These endothelial cells with somatic mutations underwent clonal expansion in response to the angiogenic signaling. And the burden of the mutant endothelial cells determined the ratio of dysplastic vessels with dilated lumen in the mosaicism. Notably, the vessel density was not correlated with the number of mutant endothelial cells in our mouse model. It was congruent with the insignificant association between presence or allelic burden of causative gene mutation and human bAVM lesion size [30]. These previous findings suggested that the aberrant endothelium was critical to induce the vascular dysplasia, while the extent of angiogenesis or lesion size was not determined by the burden of endothelium mutation alone.

In the present study, transient depletion of CNS resident macrophages in the early stage of bAVM development reduced the extent of angiogenesis via measuring the average vessel density, suggesting a more crucial crosstalk between mutant endothelial cells and immune cells during the early development of bAVM in determining the ultimate size of angiogenic lesion. Our previous data shown that the angiogenesis extent would affect not only the lesion size but also the hemorrhage of bAVM [31]. Although the angiogenic extent could not be rescued in a fully formed bAVM through transient depletion of CNS resident macrophages, other bAVM phenotypes were attenuated, such as hemorrhage, impairment of vascular integrity and morphology. Therefore, the pharmacological depletion of resident macrophage might be a promising therapy for bAVM.

Persistent inflammation has been observed in bAVMs, which accompanies dysfunctional angiogenesis. Myeloid immune cells expressing Iba1 were identified as major inflammatory components in human bAVM. The increased Iba1^+^ cells were composed of two clusters: the P2RY12^−^ perivascular macrophage and the P2RY12^+^ microglia predominantly in adjacent brain of bAVM. Previous data has demonstrated a good correlation between the expression of Iba1 and Cx3cr1 in cells localized within perivascular space and brain parenchyma [32, 23], indicating that Cx3cr1 may also be an optimal marker for CNS resident macrophages, including both microglia and perivascular macrophage. The fate mapping analysis of these brain Cx3cr1^+^ cells confirmed their common prenatal origin and their continuous residence in CNS since embryo [32, 19]. These cells subsequently differentiate into a range of CNS resident macrophages in specific niches: microglia, perivascular, meningeal, and choroid plexus macrophages [19, 32]. However, there is also evidence that inflammation in bAVM involves more than just resident immune cells. Our previous study revealed that both the circulating monocyte-derived macrophage (Ccr2^+^) and resident microglia (Cx3cr1^+^) are abundantly infiltrated and persistently activated in bAVM [16]. The increased level of monocyte-derived macrophages was even more prominent than that of resident microglia. These findings raised question regarding the extent to which the unresolved inflammation could be ascribed to, the CNS resident macrophages or the circulating monocytes. CD34^+^ peripheral blood cells of patient with *ALK1* or *ENG* gene mutation are more likely to differentiate into activated myeloid immune cells [16]. In the present study, we revealed that the CNS resident macrophages play a major role in recruiting both resident and monocyte-derived macrophages into bAVMs and promoting bAVM progression. The CSF1R inhibitors have been shown to be effective in eliminating Iba1^+^ and Cx3cr1^+^ cells present in both the parenchyma and perivascular space of CNS, which represent microglia and perivascular resident macrophages, respectively but not impede the influx of circulating monocytes into the brain [23]. In this study, we shown that depletion of CNS resident macrophages reduced the recruitment of Ccr2^+^ circulating monocyte at the early stage of bAVM development. Depletion of CNS resident macrophages transiently after bAVM has formed did not prevent the influx of Ccr2^+^ cell into the bAVM, but reduced hemorrhage and vascular dysplasia, suggesting that CNS resident macrophages play a central role in inducing peripheral monocytes into bAVMs and enhancing bAVM hemorrhage. Thus, transiently depletion of CNS resident macrophage could be development into a therapy to reduce bAVM progression and hemorrhage.

CSF1R inhibitors have served as effective tools for investigating the interplay of peripheral and CNS resident myeloid population. These oral administrated small-molecule inhibitors achieve robust but reversible brain-wide resident macrophage elimination without reactive inflammatory response or cytokine storm [33, 34]. Remarkably, pexidartinib (PLX3397) has been approved by FDA for the treatment of tenosynovial giant cell tumor in 2019 and is already in clinical trials for CNS tumors [35]. PLX5622 used in this study, has the same potency as its predecessor in CSF1R inhibition, but is 10-fold more selective in binding with the receptors and with better brain penetrance [34]. This highly selective brain penetrant CSF1R inhibitor can consistently deplete over 90% of microglia after a 7 day-treatment [24]. A sustained treatment of PLX5622 for 24 weeks is well-tolerated in rodents [34]. Upon cessation of PLX5622 treatment, the repopulation of microglia occurred within 24 hours and returned to the normal level in 36 hours [33]. The present study suggested a prolonged impact of PLX5622 in preventing bAVM progression, which lasted after microglia have repopulated. Remarkably, the efficacy of PLX5622 in elimination of resident macrophages in bAVM was not as effective as that in normal brain area, suggesting a consistent infiltration of macrophages in bAVMs despite the 7-day PLX5622 treatment. Macrophage recruitment is predisposed by *ALK1* or *Eng* gene dysfunction as indicated in our RNAseq data. It is important to determine whether an extended treatment period could further improve therapeutic effects of PLX5622 in bAVM, and the long-term outcome after treatment ceased in further study.

Notably, our present findings were not based on the assumption that CSF1R inhibitor has minimal effects on peripheral immune cells. There are disputes that the population of peripheral circulating and bone-marrow myeloid cells could also be affected by PLX5622 treatment [36]. The peripheral circulating and bone-marrow derived myeloid cells rebound at 7 days after PLX5622 cessation [36]. Monocyte-derived macrophage in CNS lesions can present competitively increase after resident population elimination with CSF1R inhibitor [18]. In the present study, infiltration of Ccr2^+^ cells within bAVM lesion were not affected by CSF1R inhibitor in the established bAVMs, which is different from those observed in the peripheral blood and bone marrow of healthy wild-type mice. Moreover, we did not find a rebound or competitive increase of Ccr2^+^ cells after PLX5622 treatment. The mice treated transiently with PLX5622 at the early stage of bAVM development reduced Ccr2^+^ cells in bAVMs analyzed 6 weeks after PLX5622 treatment stopped. The improvement of vascular phenotypes was comparable between mice with PLX5622 treatment at the early stage of bAVM development and after bAVMs had established. Therefore, the beneficial effect of CSF1R inhibition in bAVM severity appears to be independent of Ccr2^+^ cell infiltration. We also showed that the number of CNS resident macrophages was positively correlated with the number of dysplasia vessels in bAVMs. Taken together, our data shew that CNS resident macrophages may be more detrimental for bAVM progression than circulating monocytes.

**Figure 8:**
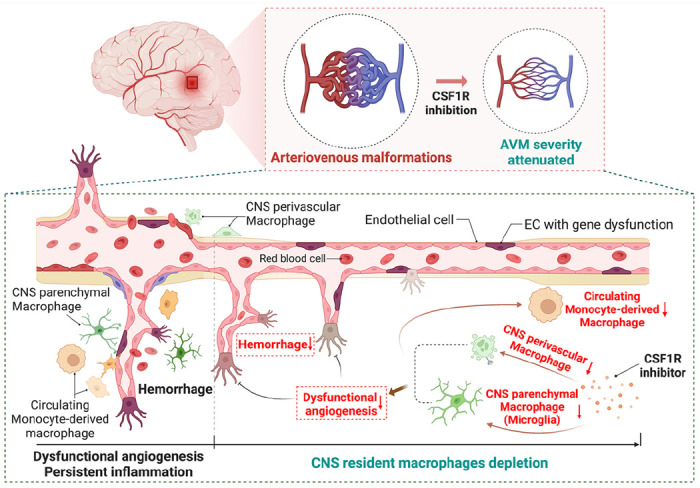
Summary of interaction between CNS resident and circulating monocyte derived macrophages in bAVM development and hemorrhage. Accumulation of CNS resident and circulating macrophages in bAVM cause dysfunctional angiogenesis and persistent inflammation which increase the risk of hemorrhage. Transient depletion of CNS resident macrophages through inhibition of CSFR1 reduced dysfunctional angiogenesis, burden of CNS resident macrophages including perivascular macrophages and microglia and circulating monocyte-derived macrophages, as well as incident of severe hemorrhage.

In summary (Fig. 8), the present study revealed that genetic dysfunction of *ALK1* or *Eng* predisposed a pro-inflammatory and pro-angiogenic microenvironment for abnormal vascular development. The CNS resident macrophages, including microglia and other non-parenchymal macrophages, play fundamental roles in orchestrating dysfunctional angiogenesis and persistent recruitment of both circulating monocytes and more resident macrophages into bAVMs promoting bAVM progression. A modulation of CNS resident macrophage would redice the vascular aberrancy, restore vascular integrity, and prevent severe hemorrhage of bAVMs.

## Data Availability

Additional Date will be made available on reasonable request.
